# High SNHG expression may predict a poor lung cancer prognosis based on a meta-analysis

**DOI:** 10.1186/s12885-023-11706-4

**Published:** 2023-12-16

**Authors:** Guo-yi Shen, Rong-zhi Huang, Shao-bin Yang, Rong-qiang Shen, Jian-li Gao, Yi Zhang

**Affiliations:** Department of Cardiothoracic Surgery, Zhangzhou Affiliated Hospital of Fujian Medical University, No. 59, Shengli Road, Zhangzhou City, Zhangzhou, Fujian 363000 China

**Keywords:** lncRNA, SNHG, Lung cancer, Prognosis, Meta-analysis

## Abstract

**Background:**

An increasing number of small nucleolar RNA host genes (SNHGs) have been revealed to be dysregulated in lung cancer tissues, and abnormal expression of SNHGs is significantly correlated with the prognosis of lung cancer. The purpose of this study was to conduct a meta-analysis to explore the correlation between the expression level of SNHGs and the prognosis of lung cancer.

**Methods:**

A comprehensive search of six related databases was conducted to obtain relevant literature. Relevant information, such as overall survival (OS), progression-free survival (PFS), TNM stage, lymph node metastasis (LNM), and tumor size, was extracted. Hazard ratios (HRs) and 95% confidence intervals (CIs) were pooled to evaluate the relationship between SNHG expression and the survival outcome of lung cancers. Sensitivity and publication bias analyses were performed to explore the stability and reliability of the overall results.

**Results:**

Forty publications involving 2205 lung cancer patients were included in this meta-analysis. The pooled HR and 95% CI values indicated a significant positive association between high SNHG expression and poor OS (HR: 1.890, 95% CI: 1.595–2.185), disease-free survival (DFS) (HR: 2.31, 95% CI: 1.57–3.39) and progression-free survival (PFS) (HR: 2.01, 95% CI: 0.66–6.07). The pooled odds ratio (OR) and 95% CI values indicated that increased SNHG expression may be correlated with advanced TNM stage (OR: 1.509, 95% CI: 1.267–1.799), increase risk of distant lymph node metastasis (OR: 1.540, 95% CI: 1.298–1.828), and large tumor size (OR: 1.509, 95% CI: 1.245–1.829). Sensitivity analysis and publication bias results showed that each result had strong reliability and robustness, and there was no significant publication bias or other bias.

**Conclusion:**

Most SNHGs are upregulated in lung cancer tissues, and high expression of SNHGs predicts poor survival outcomes in lung cancer. SNHGs may be potential prognostic markers and promising therapeutic targets.

**Supplementary Information:**

The online version contains supplementary material available at 10.1186/s12885-023-11706-4.

## Introduction

Cancer is a major threat to human health [1, 2]. Thousands of people die of cancer every year, which brings an enormous economic burden to the whole world [3]. According to reports, in 2020, there was an estimated 19.8 million new cancer cases and nearly 10 million cancer deaths worldwide [2]. Among cancers, lung cancer ranks first in incidence among men and second among women [4, 5]. Various treatment modalities, such as surgery, radiotherapy, chemotherapy, targeted therapy and immunotherapy, have been applied for cancer treatment, and patient survival has improved. However, many patients are already in the middle and advanced stages of the disease when they are diagnosed [6], and the five-year survival rate of lung cancer is still not optimistic [7, 8]. An increasing number of researchers are trying to find new therapeutic targets [9–11].

With the advancement of cancer research at the level of molecular biology, long noncoding RNAs have been reported by many scientists to be significant factors in the progression of lung cancer [12–14]. Although they have no protein coding ability, long noncoding RNAs can directly act on downstream genes or signaling pathways and intervene in the proliferation, migration, invasion and drug resistance of lung cancer cells [15]. For instance, Guo et al. uncovered that linc00261 could suppress the proliferation, migration and invasion of lung cancer cells by increasing FOXO1 expression by downregulating miR-1269a [16]. Xu et al. revealed that linc00473 contributes to the invasion, migration and proliferation of NSCLC cells by sponging and downregulating miR-497-5p [17].

The expression of many SNHGs has been revealed by researchers to be dysregulated in lung cancer tissues and to be closely involved in the occurrence and development of lung cancer. SNHG can directly regulate the downstream genes or signaling pathways of lung cancer cells or act as a molecular sponge of microRNAs and then indirectly regulate downstream signaling cascades to affect the proliferation, migration, invasion and apoptosis of tumor cells [18, 19]. For instance, Wang et al. suggested that SNHG12 promotes the migration and invasion of NSCLC cells by interacting with the Slug/ZEB2 signaling pathway by serving as a sponge of miR-218 [20]. Zhao et al. discovered that SNHG3 facilitates the invasion, proliferation, and migration and inhibits the apoptosis of NSCLC cells through the upregulation of nuclear factor IX (NFIX) by sponging and downregulating miR-1343-3p [21]. An increasing number of studies have reported that SNHGs are upregulated and significantly related to the prognosis of lung cancer, while other studies have obtained the opposite results. Considering that the sample size of single studies on this topic are insufficient, and the conclusions of different studies are not completely consistent, the purpose of this study was to conduct a meta-analysis to comprehensively explore the correlation between the expression level of SNHG and the prognosis of lung cancer.

## Materials and methods

### Literature search strategy

Based on the Preferred Reporting Items for Systematic Reviews and Meta-Analyses, a comprehensive search of six related electronic databases, including PubMed, Embase, Web of Science, Cochrane Library, Google Scholar and China National Knowledge Infrastructure (CNKI), was performed. The detailed search terms were as follows: (“small nucleolar RNA host gene” OR “Long noncoding RNA SNHG” OR “SNHG” OR “lnc SNHG”) AND (“non-small cell lung cancer” OR “lung cancer” OR “Lung adenocarcinoma” OR “NSCLC” OR “prognosis” OR “survival” OR “outcome”). The references of the included literature were also read in detail to avoid omitting relevant literature as much as possible.

### Inclusion and exclusion criteria

Based on the reporting specification of The Preferred Reporting Items for Systematic Reviews and Meta-Analyses (PRISMA) for meta-analysis, the original documents included in this study met the following inclusion and exclusion criteria. Inclusion criteria: (1) There is a clear detection method to detect the expression level of SNHG in tumor tissues, such as real-time fluorescence quantitative polymerase chain reaction (qRT‒PCR). (2) Based on the expression level of SNHG, patients were divided into a high-expression SNHG group and a low-expression SNHG group. (3) The literature mainly evaluates the correlation between the expression level of SNHG and the prognosis of lung cancer. (4) The original documents provide sufficient data for statistics. Exclusion criteria: (1) The original literature did not evaluate the correlation between the expression level of SNHG and the prognosis of lung cancer. (2) Insufficient or unavailable data. (3) The research objects are not humans but animals. (4) Written in a language other than English.

### Quality evaluation of included literature

For each publication included in this meta-analysis, the quality assessment according to the Newcastle‒Ottawa Scale (NOS) score was independently conducted by two researchers, which included three major items: selection method of case group and control group, comparability of case group and control group, and contact exposure assessment method. According to the star rating system, the three items have a total of 9 points; the lower the score is, the worse the quality of the literature research. The literature with a score below 6 will be excluded, and the literature with a score of 6–9 is considered suitable for inclusion in this study [22].

### Data extraction

Two researchers independently obtained usable or related data, such as the name of the first author, year of publication, sample size, cutoff value, detection methods, and follow-up month. The number of occurrences and the total number of events were extracted to evaluate the correlation between SNHG expression levels and various clinicopathological features, such as TNM stage, LNM, DM, tumor size, and histological grade. Hazard ratios (HRs) with 95% confidence intervals (CIs) were obtained to evaluate the relationship between SNHG expression and the survival outcomes of lung cancer, including OS, progression-free survival (PFS), disease-free survival (DFS) and relapse-free survival (RFS). If the literature did not directly give the HR value but contained a survival curve and the number of people with high and low expression of SNHG, we obtained the HR value and its 95% confidence interval according to the software Enguage version 4.0 [23].

### Statistical analysis

Stata version 12.0 software (Stata Corporation, College Station, TX) and Review Manager 5.4.0 (Cochrane Collaboration) were utilized in this meta-analysis. Pooling HR with 95% CI was carried out to assess the association between SNHG expression and cancer prognosis. Pooling OR with 95% CI was performed to explore the relationship between SNHG expression and clinicopathological features of lung cancers. For the heterogeneity of each result, the fix-effect model was performed for small heterogeneity (*I*^2^ < 50%, *p* ≥ 0.05). If the heterogeneity was significant (*I*^2^ ≥ 50%, *p* < 0.05), the random-effect model was used, and subgroup analysis was conducted based on the SNHG expression, follow-up month, number of patients, NOS score and so on.

## Results

### The basic characteristics of the included studies

**Fig. 1:**
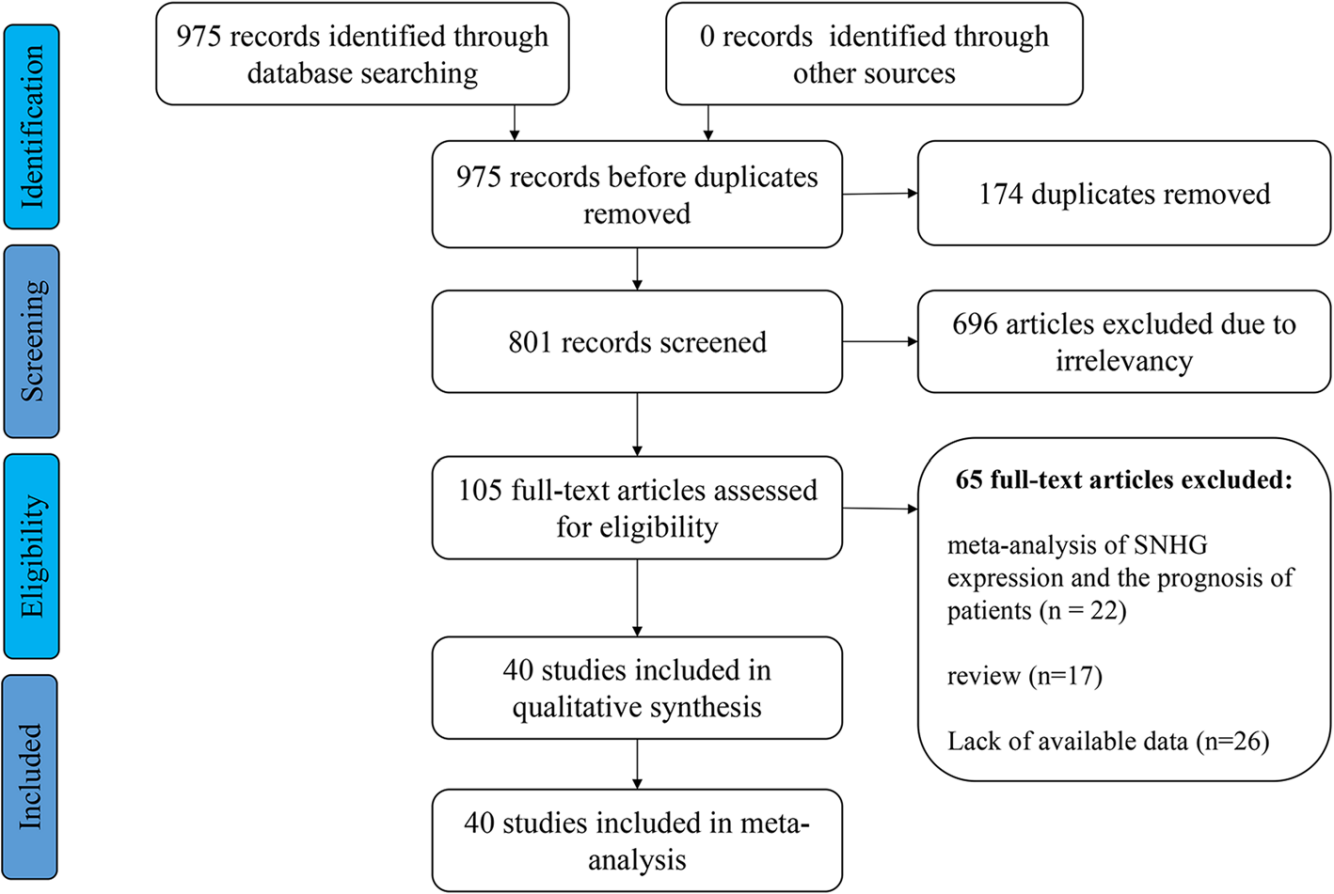
The flow diagram of the eligible studies

After the comprehensive search of related databases, 975 articles were initially obtained, 174 duplicate publications were excluded, and 696 studies were discarded because they did not assess the correlation between the expression level of SNHG and the prognosis of lung cancer. In addition, 22 meta-analyses, 17 reviews and 26 articles with insufficient data were removed. Finally, 40 studies with 2205 lung cancer patients were enrolled in this meta-analysis [20, 21, 24–60] (Fig. 1). All patients were from China, and the expression level of SNHG was mostly detected by clear detection methods, such as real-time fluorescent quantitative polymerase chain reaction (qRT‒PCR), and there were clear reference genes (Table 1). Based on the NOS scale, the research quality of the 41 original documents was no less than 6 points (Table 2).

**Table 1:** Basic features of the publications included in this meta-analysis (*n* = 40)

Author and year	Sample size	Level of expression	Detection methods	Cut-off value	Reference gene	Survival outcome	HR with 95%CI	LCI	UCI	Analysis methods	Follow-up (month)	NOS score
Wei L 2019	64	upregulated	qRT-PCR	median	GAPDH	OS	2.56	1.2	5.47	Univariate analysis	24	8
Cui Y 2017	68	upregulated	qRT-PCR	median	GAPDH	OS	2.07	1.13	3.79	Univariate analysis	60	8
Li XM 2020	40	upregulated	qRT-PCR	mean	GAPDH	Not reported	-	-	-	-	-	7
Zhang HY 2017	36	upregulated	qRT-PCR	mean	GAPDH	Not reported	-	-	-	-	-	7
Li CL 2019	64	upregulated	qRT-PCR	mean	GAPDH	Not reported	-	-	-	-	-	7
Yang XH 2021	41	downregulated	qRT-PCR	mean	GAPDH	Not reported	-	-	-	-	-	7
Kang BJ 2021	66	downregulated	qRT-PCR	mean	GAPDH	Not reported	-	-	-	-	-	7
Zhao LJ 2021	35	upregulated	qRT-PCR	median	GAPDH	Not reported	-	-	-	-	-	7
Shi JD 2019	32	upregulated	qRT-PCR	median	GAPDH	OS	2.71	0.99	7.41	Univariate analysis	60	8
Zhao SS 2020	42	upregulated	qRT-PCR	Not reported	GAPDH	OS	2.16	0.73	6.38	Univariate analysis	60	7
Wang F 2021	50	upregulated	qRT-PCR	mean	GAPDH	OS	1.79	0.67	4.8	Univariate analysis	60	8
						DFS	2.03	0.97	4.27	Univariate analysis	60	8
Gao N 2020	45	upregulated	qRT-PCR	mean	GAPDH	OS	2.34	1.19	4.61	Univariate analysis	60	6
Dong Z 2020	40	upregulated	qRT-PCR	mean	GAPDH	OS	2.36	0.9	6.17	Univariate analysis	60	6
Geng H 2020	60	upregulated	qRT-PCR	median	GAPDH	OS	1.76	0.8	3.86	Univariate analysis	36	8
Liang R 2018	60	upregulated	qRT-PCR	mean	GAPDH	OS	2.33	1.23	4.40	Univariate analysis	48	8
Pang LL 2019	42	upregulated	qRT-PCR	mean	GAPDH	Not reported	-	-	-	-	-	7
Li LP 2020	36	upregulated	qRT-PCR	mean	GAPDH	OS	3.85	1.86	10.96	Univariate analysis	60	6
Chen CH 2018	120	upregulated	qRT-PCR	median	β-actin	OS	1.3	0.88	1.9	Univariate analysis	60	8
						PFS	1.24	0.85	1.79	Univariate analysis	60	8
Wang RX 2020	50	upregulated	qRT-PCR	mean	GAPDH	OS	2.55	0.85	7.66	Univariate analysis	48	6
Liu SX 2019	42	upregulated	qRT-PCR	median	GAPDH	OS	2.07	1.04	4.14	Univariate analysis	60	8
Huang YS 2022	65	upregulated	qRT-PCR	mean	GAPDH	OS	2.25	1.05	4.86	Univariate analysis	60	8
Wang Y 2019	40	upregulated	qRT-PCR	median	GAPDH	OS	2.27	0.83	6.19	Univariate analysis	48	6
Wang S 2018	128	upregulated	qRT-PCR	mean	GAPDH	OS	2.1276	1.0638	4.1667	Univariate analysis	60	6
Guo LF 2018	40	upregulated	qRT-PCR	mean	GAPDH	OS	-	-	-	-	-	7
Huang YF 2021	68	upregulated	qRT-PCR	mean	GAPDH	OS	4.09	1.94	8.61	Univariate analysis	48	8
Zhang NN 2021	45	upregulated	qRT-PCR	mean	GAPDH	OS	2.27	0.69	7.41	Univariate analysis	54	8
Chen XL 2020	50	upregulated	qRT-PCR	mean	GAPDH	OS	2.11	0.9	4.96	Univariate analysis	60	8
Zhang ZH 2018	99	upregulated	qRT-PCR	mean	GAPDH	OS	1.66	0.94	2.93	Univariate analysis	60	8
Ma XR 2019	24	upregulated	qRT-PCR	mean	GAPDH	OS	3.298	1.3635	7.9774	Univariate analysis	60	6
Dong YZ 2018	49	upregulated	qRT-PCR	median	GAPDH	OS	1.84	0.94	3.59	Univariate analysis	120	8
						DFS	2.22	1.17	4.19	Univariate analysis	80	8
Cui HX 2018	55	upregulated	qRT-PCR	mean	GAPDH	OS	2.234	1.0334	4.8294	Univariate analysis	80	8
Han PF 2021	118	upregulated	qRT-PCR	mean	GAPDH	OS	3.71	1.67	6.94	9	60	
Jin B 2018	35	upregulated	qRT-PCR	median	GAPDH	OS	1.63	0.56	4.76	Univariate analysis	60	8
Li Y 2022	30	upregulated	qRT-PCR	mean	GAPDH	Not reported	-	-	-	-	-	7
Han W 2018	66	upregulated	qRT-PCR	mean	β-actin	OS	2.73	1.381	5.397	Multivariate analysis	60	9
						DFS	2.641	1.394	5.002	Multivariate analysis	60	9
Zhang ZW 2021	50	upregulated	qRT-PCR	median	GAPDH	OS	2.2	0.93	5.19	Univariate analysis	120	8
Fan HJ 2021	63	upregulated	qRT-PCR	mean	GAPDH	OS	2.459	1.584	4.971	Multivariate analysis	60	9
Jin LL 2019	42	upregulated	qRT-PCR	median	GAPDH	OS	2.46	0.96	6.29	Univariate analysis	60	6
Chen ZY 2017	42	upregulated	qRT-PCR	median	GAPDH	OS	6.06	2.44	15.07	Univariate analysis	24	8
						PFS	3.89	1.48	10.22	Univariate analysis	24	8
Wang XL 2020	63	upregulated	qRT-PCR	mean	GAPDH	Not reported	-	-	-	-		7

**Table 2:** Quality assessment of eligible studies Newcastle–Ottawa scale (NOS) score

Author	Country	**Selection**				**Comparability**	**Outcome**			**Total**
		Adequate of case definition	Representativeness of the cases	Selection of Controls	Definition of Controls	Comparability of cases and controls	Ascertainment of exposure	Same method of ascertainment	Non-Response rate	
Wei L 2019	China	*	*	*	*	*	*	*	*	8^a^
Cui Y 2017	China	*	*	*	*	*	*	*	*	8^a^
Li XM 2020	China	*	*	*	*	*	-	*	*	7^c^
Zhang HY 2017	China	*	*	*	*	*	-	*	*	7^c^
Li CL 2019	China	*	*	*	*	*	*	*	-	7^c^
Yang XH 2021	China	*	*	*	*	*	*	*	-	7^c^
Kang BJ 2021	China	*	*	*	*	*	*	*	-	7^c^
Zhao LJ 2021	China	*	*	*	*	*	*	*	-	7^c^
Shi JD 2019	China	*	*	*	*	*	*	*	*	8^a^
Zhao SS 2020	China	*	*	*	*	*	*	*	-	7^b^
Wang F 2021	China	*	*	*	*	*	*	*	*	8^a^
Gao N 2020	China	*	*	*	*	*	*	-	-	6^ab^
Dong Z 2020	China	*	*	*	*	*	-	*	-	6^abd^
Geng H 2020	China	*	*	*	*	*	*	*	*	8^a^
Liang R 2018	China	*	*	*	*	*	*	*	*	8^a^
Pang LL 2019	China	*	*	*	*	*	*	*	-	7^c^
Li LP 2020	China	*	*	*	*	*	-	*	-	6^bd^
Chen CH 2018	China	*	*	*	*	*	*	*	*	8^a^
Wang RX 2020	China	*	*	*	*	*	-	*	-	6^bd^
Liu SX 2019	China	*	*	*	*	*	*	*	*	8^a^
Huang YS 2022	China	*	*	*	*	*	*	*	*	8^a^
Wang Y 2019	China	*	*	*	*	*	*	-	-	6^bd^
Wang S 2018	China	*	*	*	*	*	-	*	-	6^bd^
Guo LF 2018	China	*	*	*	*	*	*	*	-	7^c^
Huang YF 2021	China	*	*	*	*	*	*	*	*	8^a^
Zhang NN 2021	China	*	*	*	*	*	*	*	*	8^a^
Chen XL 2020	China	*	*	*	*	*	*	*	*	8^a^
Ma XR 2019	China	*	*	*	*	*	*	*	*	8^a^
Dong YZ 2018	China	*	*	*	*	*	-	*	-	6^ad^
Cui HX 2018	China	*	*	*	*	*	*	*	*	8^a^
Han PF 2021	China	*	*	*	*	**	*	*	*	9
Jin B 2018	China	*	*	*	*	*	*	*	*	8^a^
Li Y 2022	China	*	*	*	*	*	*	*	-	7^cd^
Han W 2018	China	*	*	*	*	**	*	*	*	9
Zhang ZW 2021	China	*	*	*	*	*	*	*	*	8^a^
Fan HJ 2021	China	*	*	*	*	**	*	*	*	9
Jin LL 2019	China	*	*	*	*	*	-	*	-	6^bd^
Chen ZY 2017	China	*	*	*	*	*	*	*	*	8^a^
Wang XL 2020	China	*	*	*	*	*	*	*	-	7^cd^

^a^Only univariate analysis was performed, and data for multivariate analysis were lacking

^b^The article only provides data on survival prognosis and does not provide clinical pathological parameters

^c^Lack of data on survival prognosis, only clinicopathological parameters were provided

^d^Fewer cases

### The association between SNHG expression and overall survival

**Fig. 2:**
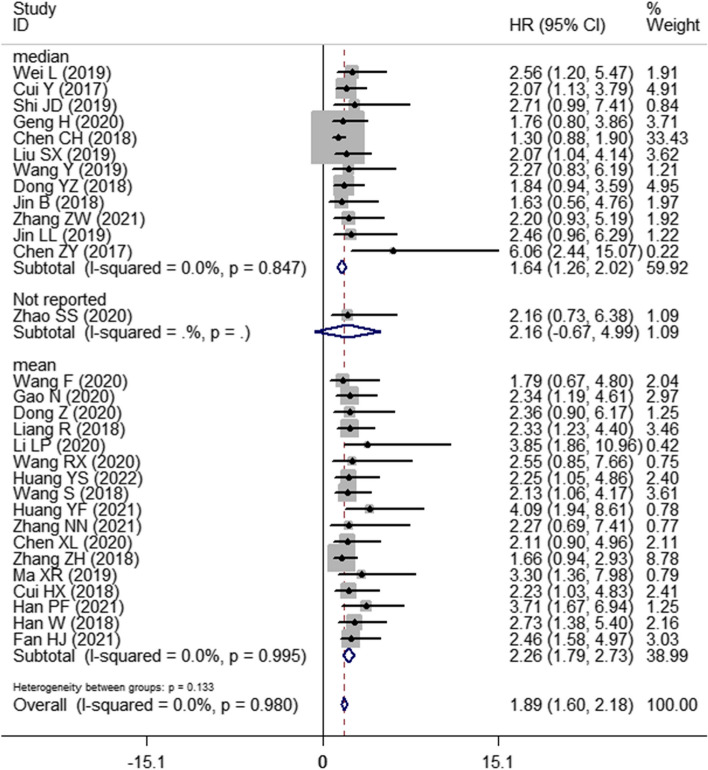
Forest plot showing the relationship between SNHG expression and overall survival (OS) in lung cancers

**Fig. 3:**
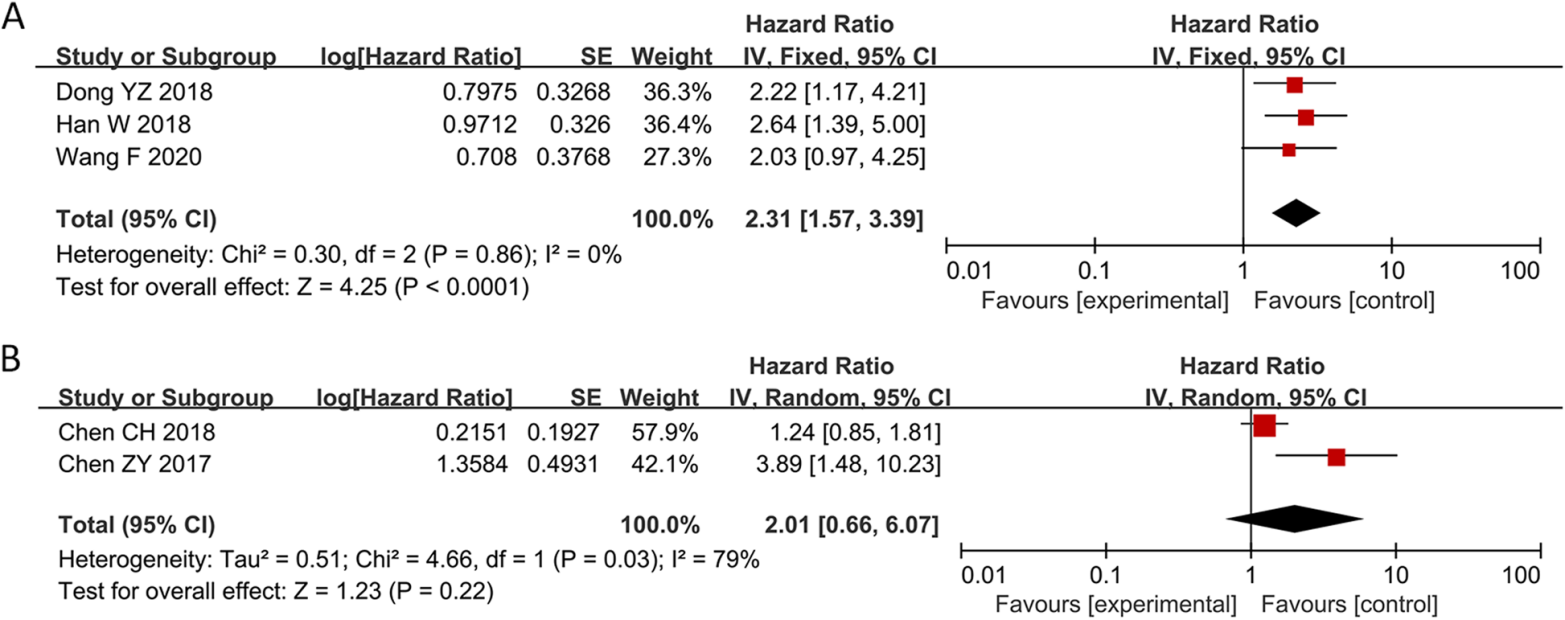
Forest plot showing the relationship between SNHG expression and disease-free survival and progression-free survival. Note: **A** DFS, **B** PFS

Thirty studies with 1748 patients were enrolled to assess the association between SNHG expression and the prognosis of lung cancer. The pooled HR with 95% CI indicated a significant positive relationship between high SNHG expression and poor OS (HR: 1.890, 95% CI: 1.595–2.185) (Fig. 2). In consideration of the inconsistent cutoff values, follow-up time, NOS scores and HR sources between different primary studies, the results of the subgroup analysis show that elevated SNHG expression implies worse OS in the mean value cutoff subgroup (HR: 2.262, 95% CI: 1.790–2.734), the median-value cutoff subgroup (HR: 1.643, 95% CI: 1.262–2.024), the subgroup of multivariate analysis (HR: 2.793, 95% CI: 1.631–3.955) and univariate analysis (HR: 1.828, 95% CI: 1.523–2.133). At the same time, the pooled HR and 95% CI values revealed that high SNHG expression predicted poor disease-free survival (DFS) (HR: 2.31, 95% CI: 1.57–3.39) (Fig. 3A) and progression-free survival (PFS) (HR: 2.01, 95% CI: 0.66–6.07) (Fig. 3B and Table 3).

**Table 3:** Pooled HRs of overall survival of patients with increased SNHG expression

	No. of studies	No. of patients	Pooled HR (95% CI)		Heterogeneity	
			Fixed	Random	*I*^2^(%)	*P*-value
**OS**	30	1748	1.890 (1.595–2.185)	1.89 (1.595–2.185)	0	0.98
**Cut-off value**						
Median	12	644	1.643 (1.262–2.024)	1.643 (1.262–2.024)	0	0.847
Mean	17	1062	2.262 (1.790–2.734)	2.262 (1.790–2.734)	0	0.995
Not reported	1	42	2.160 (-0.665–4.985)	2.160 (-0.665–4.985)	-	-
**Analysis method**						
Multivariate analysis	3	247	2.793 (1.631–3.955)	2.793 (1.631–3.955)	0	0.734
Univariate analysis	27	1501	1.828 (1.523–2.133)	1.828 (1.523–2.133)	0	0.988
**Number of patients**						
Less than 100	27	1382	2.159 (1.783–2.534)	2.159 (1.783–2.534)	0	1
Not less than 100	3	366	1.457 (0.980–1.933)	1.873 (0.764–2.982)	48.6	0.143
**Follow-up (month)**						
Not less than 60 month	22	1319	1.819 (1.503–2.135)	1.819 (1.503–2.135)	0	0.962
Less than 60 month	8	429	2.373 (1.550–3.197)	2.373 (1.550–3.197)	0	0.885
**NOS score**						
9	3	247	2.793 (1.631–3.955)	2.793 (1.631–3.955)	0	0.734
Less than 9	27	1501	1.828 (1.523–2.133)	1.828 (1.523–2.133)	0	0.988

*OS* Overall survival, *Random* Random effects, *Fixed* Fixed effects, *directly* HR was extracted directly from the primary articles, *indirectly* HR was extracted indirectly from the primary articles, *CI* Confidence interval

### The association between SNHG expression and TNM stage

**Fig. 4:**
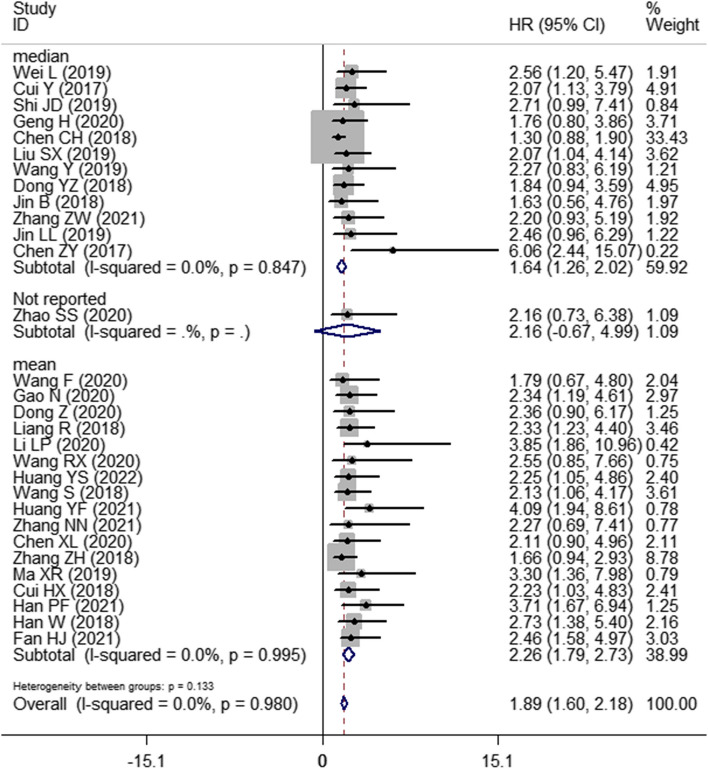
Forest plot of the relationship between SNHG expression and TNM stage in lung cancers

Twenty-eight studies comprising 1589 patients were included in this study to explore the relationship between SNHG expression and TNM stage. Pooled OR and 95% CI values showed that high SNHG expression predicted advanced TNM stage (OR: 1.509, 95% CI: 1.267–1.799) (Fig. 4). The results of subgroup analysis indicated advanced TNM stage of lung cancers was correlated with high SNHG expression (OR: 1.650, 95% CI: 1.374–1.980), NOS score ≥ 9 (OR: 2.043, 95% CI: 1.215–3.435) and NOS score < 9 (OR: 1.451, 95% CI: 1.204–1.748). However, in the low SNHG expression subgroup, high SNHG expression implies a favorable TNM stage (OR: 0.424, 95% CI: 0.207–0.867) (Table 4).

**Table 4:** Pool effects of clinicopathologic characteristics in lung cancer patients with abnormal SNHG expression

Clinicopathologic characteristics	No. of studies	No. of patients	Odds ratio (95% CI)		*P*	Heterogeneity	
			Fixed	Random		*I*^2^(%)	*P*-value
*Age*	28	1619	1.045 (0.879–1.242)	1.045 (0.879–1.243)	0.62	0	1
*Gender*	28	1584	0.970 (0.806–1.166)	0.970 (0.806–1.168)	0.744	0	1
*TNM* (**III**+**IV*** vs*. **I**+**II**)	28	1589	1.509 (1.267–1.799)	1.521 (1.220–1.897)	< 0.0001	30.3	0.064
SNHG expression							
High SNHG expression	26	1484	1.650 (1.374–1.980)	1.654 (1.366–2.003)	< 0.0001	4.2	0.402
Low SNHG expression	2	105	0.424 (0.207–0.867)	0.426 (0.208–0.872)	0.019	0	0.684
NOS score							
9	2	184	2.043 (1.215–3.435)	2.039 (1.212–3.432)	0.007	0	0.616
Less than 9	26	1405	1.451 (1.204–1.748)	1.472 (1.160–1.868)	< 0.0001	32.5	0.054
*LNM* (*present vs. absent*)	29	1662	1.540 (1.298–1.828)	1.551 (1.257–1.914)	< 0.0001	27.1	0.091
SNHG expression							
High SNHG expression	27	1557	1.681 (1.406–2.008)	1.666 (1.389–1.997)	< 0.0001	0	0.514
Low SNHG expression	2	105	0.435 (0.216–0.876)	0.437 (0.216–0.882)	0.02	0	0.613
NOS score							
9	3	247	1.857 (1.203–2.865)	1.848 (1.195–2.858)	0.005	0	0.574
Less than 9	26	1415	1.488 (1.235–1.792)	1.504 (1.187–1.905)	< 0.0001	31.4	0.065
*Tumor size* (*big vs small*)	23	1324	1.509 (1.245–1.829)	1.557 (1.217–1.992)	< 0.0001	31.7	0.074
SNHG expression							
High SNHG expression	21	1219	1.645 (1.344–2.013)	1.664 (1.325–2.091)	< 0.0001	14.9	0.264
Low SNHG expression	2	105	0.584 (0.296–1.149)	0.587 (0.293–1.176)	0.119	2.5	0.311
NOS score							
9	1	66	1.917 (0.820–4.478)	1.917 (0.820–4.478)	0.133	-	-
less than 9	22	1258	1.489 (1.222–1.814)	1.545 (1.194–2.001)	0.001	34.1	0.061
*Histological grade*	9	567	1.248 (0.938–1.661)	1.247 (0.936–1.660)	0.128	0	0.97
NOS score							
9	2	184	1.380 (0.831–2.291)	1.378 (0.828–2.292)	0.213	0	0.43
Less than 9	7	383	1.191 (0.843–1.683)	1.190 (0.842–1.683)	0.321	0	0.962
*DM* (*present vs. absent*)	3	136	0.933 (0.463–1.882)	0.933 (0.462–1.885)	0.848	0	0.929
*Invasion depth* (*T3*+*T4/T1*+*T2*)	1	40	1.029 (0.297–3.566)	1.029 (0.297–3.566)	0.965	-	-

*TNM* Tumor Node Metastasis, *LNM* Lymph node metastasis, *DM* Distant metastasis, *CI* Confidence interval, *No.* Number, *NA* Not applicable

### The association between SNHG expression and LNM

**Fig. 5:**
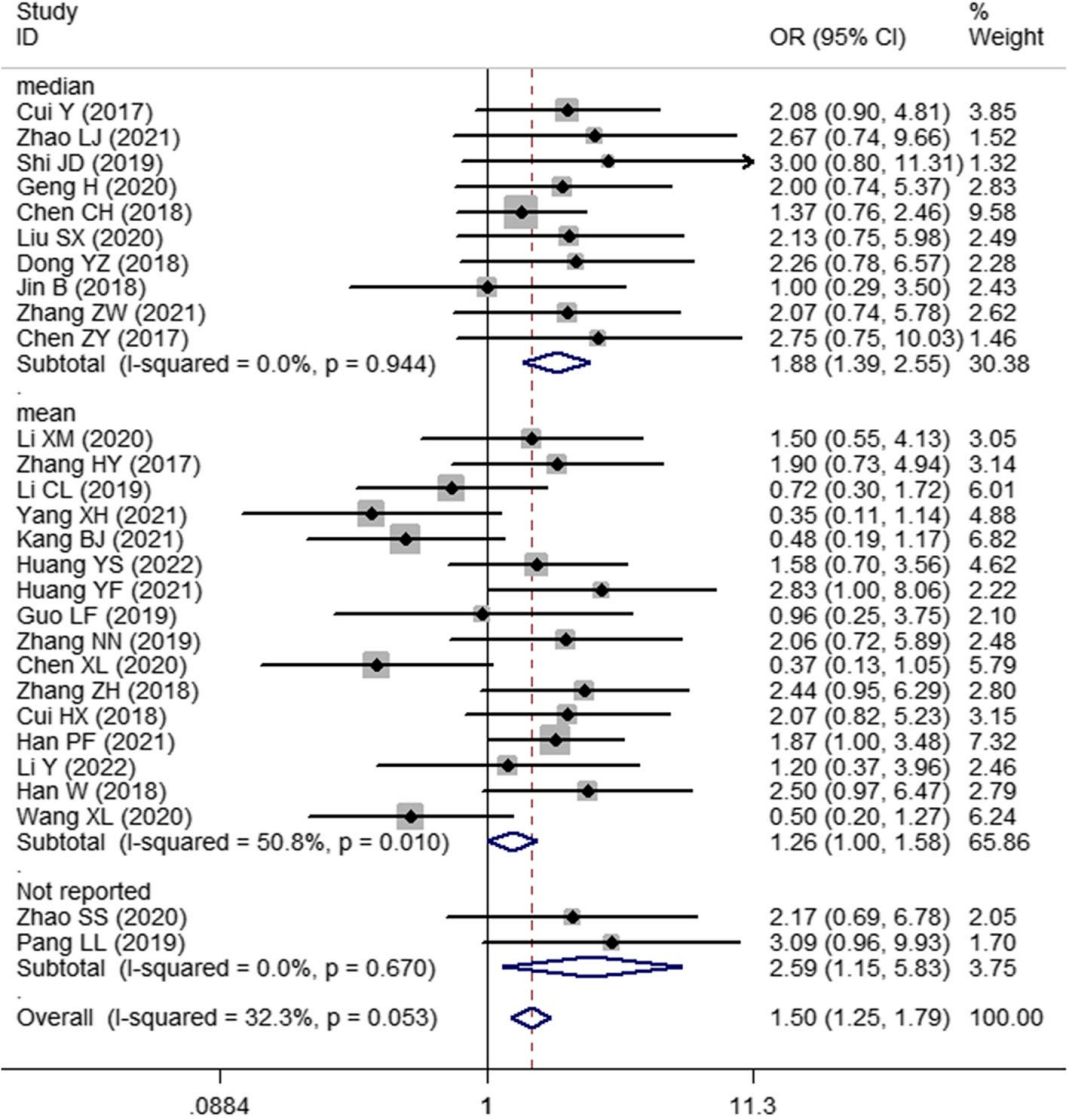
Forest plot of the relationship between SNHG expression and LNM in lung cancers

Twenty-nine publications involving 1662 patients were enrolled to evaluate the relationship between SNHG expression and LNM. Pooled OR and 95% CI results suggested a significant association between increased SNHG expression and distant lymph node metastasis (OR: 1.540, 95% CI: 1.298–1.828) (Fig. 5). Based on the subgroup analysis, we found an increased risk of distant lymph node metastasis of lung cancer cells in the subgroups with high SNHG expression (OR: 1.681, 95% CI: 1.406–2.008), NOS score ≥ 9 (OR: 1.857, 95% CI: 1.203–2.865) and NOS score < 9 (OR: 1.488, 95% CI: 1.235–1.792). In addition, there was a lower likelihood of distant lymph node metastasis in the subgroup with low SNHG expression (OR: 0.435, 95% CI: 0.216–0.876) (Table 4).

### The association between SNHG expression and other clinicopathological parameters

**Fig. 6:**
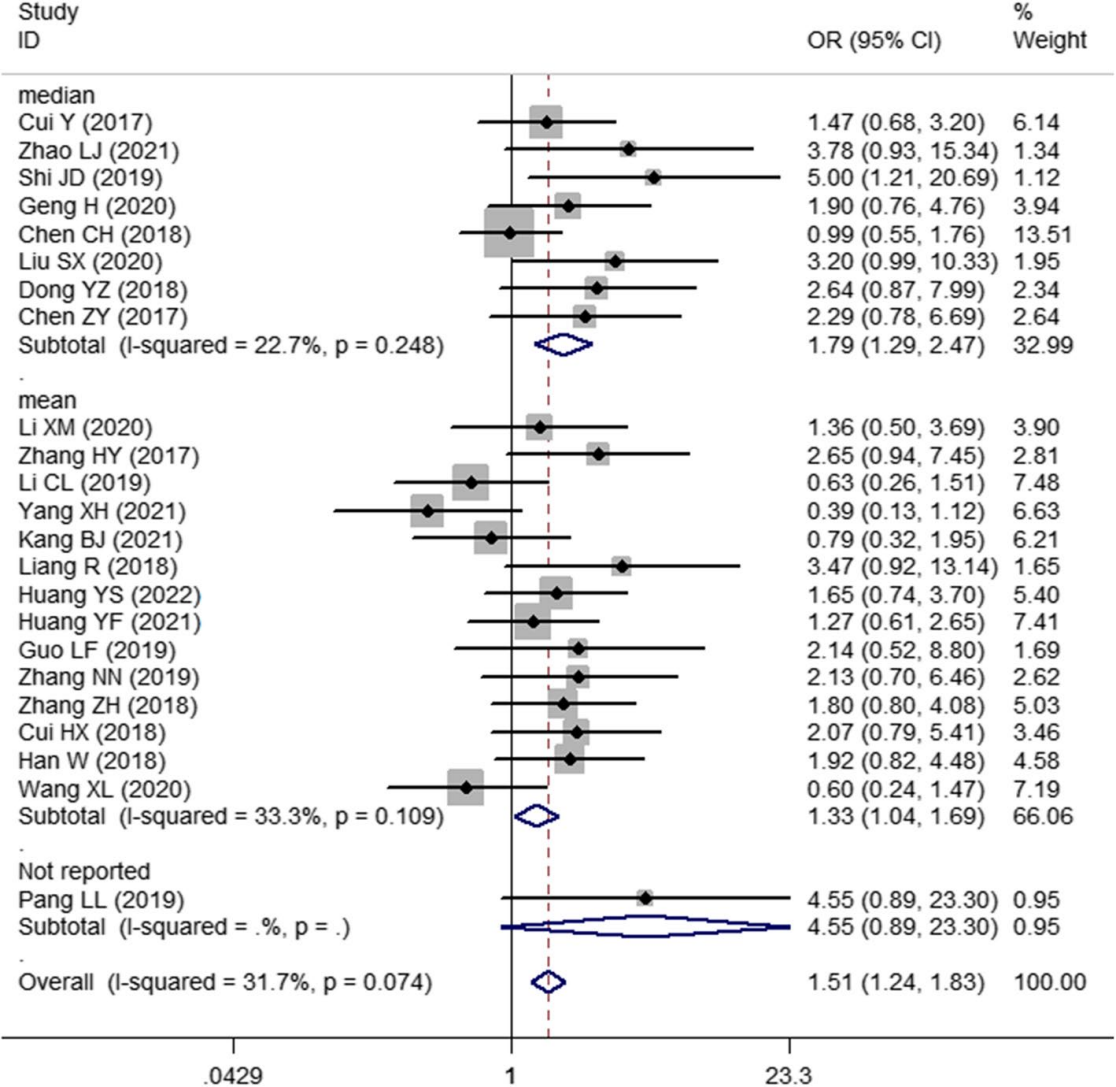
Forest plot of the relationship between SNHG expression and tumor size in lung cancers

**Fig. 7:**
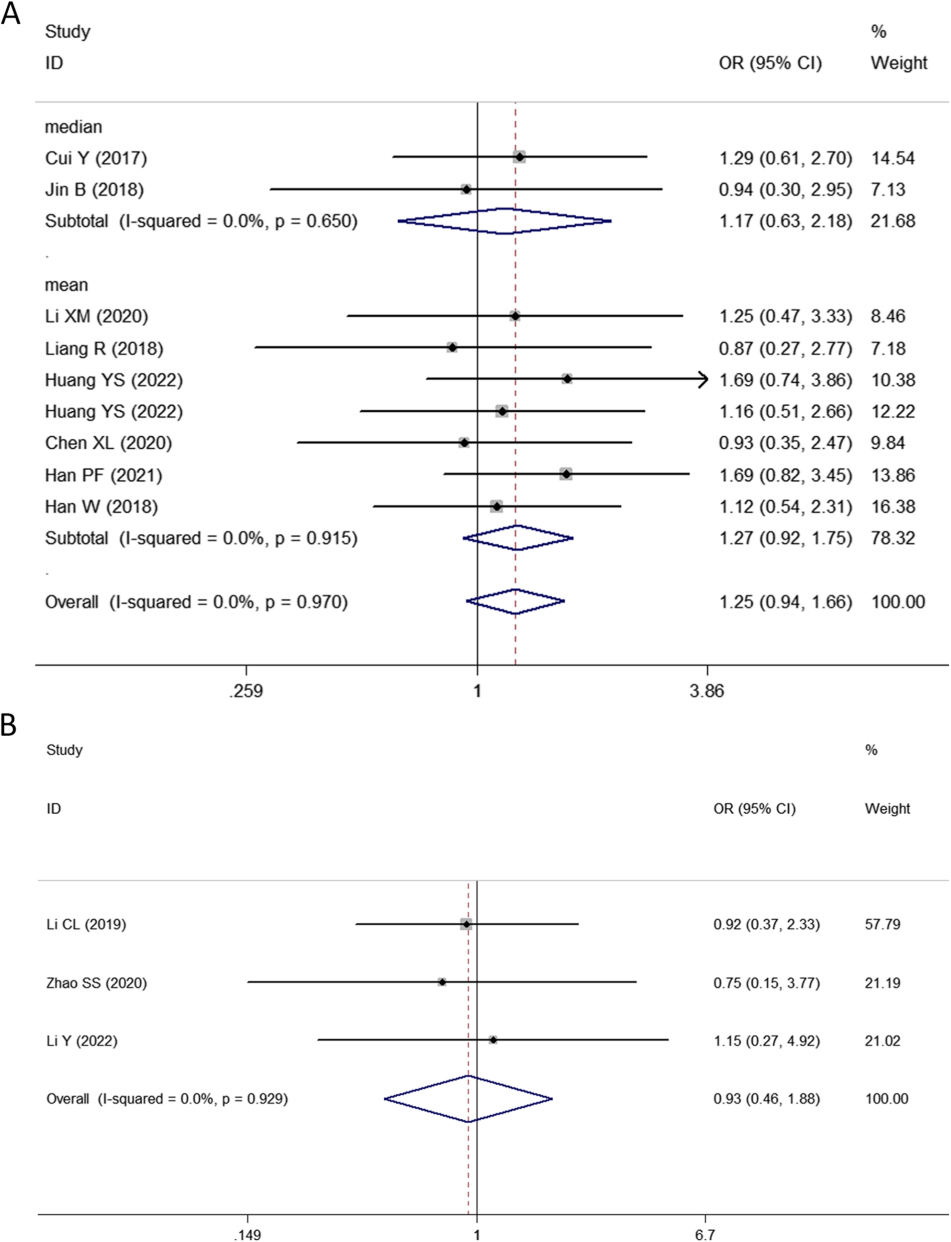
Forest plot of the relationship between SNHG expression and histological grade and distant metastasis in lung cancers. Note: **A** histological grade, **B** distant metastasis

Pooled OR and 95% CI values also revealed a marked positive correlation between high SNHG expression and large tumor size (OR: 1.509, 95% CI: 1.245–1.829) (Fig. 6). The correlations between SNHG expression and histological grade (OR: 1.248, 95% CI: 0.938–1.661) (Fig. 7A), depth of invasion (OR: 1.029, 95% CI: 0.297–3.566), DM (OR: 0.933, 95% CI: 0.463–1.882) (Fig. 7B), age (OR: 1.045, 95% CI: 0.879–1.242) and sex (OR: 0.970, 95% CI: 0.806–1.166) were nonsignificant (Table 4).

### Sensitivity and publication bias analyses

**Fig. 8:**
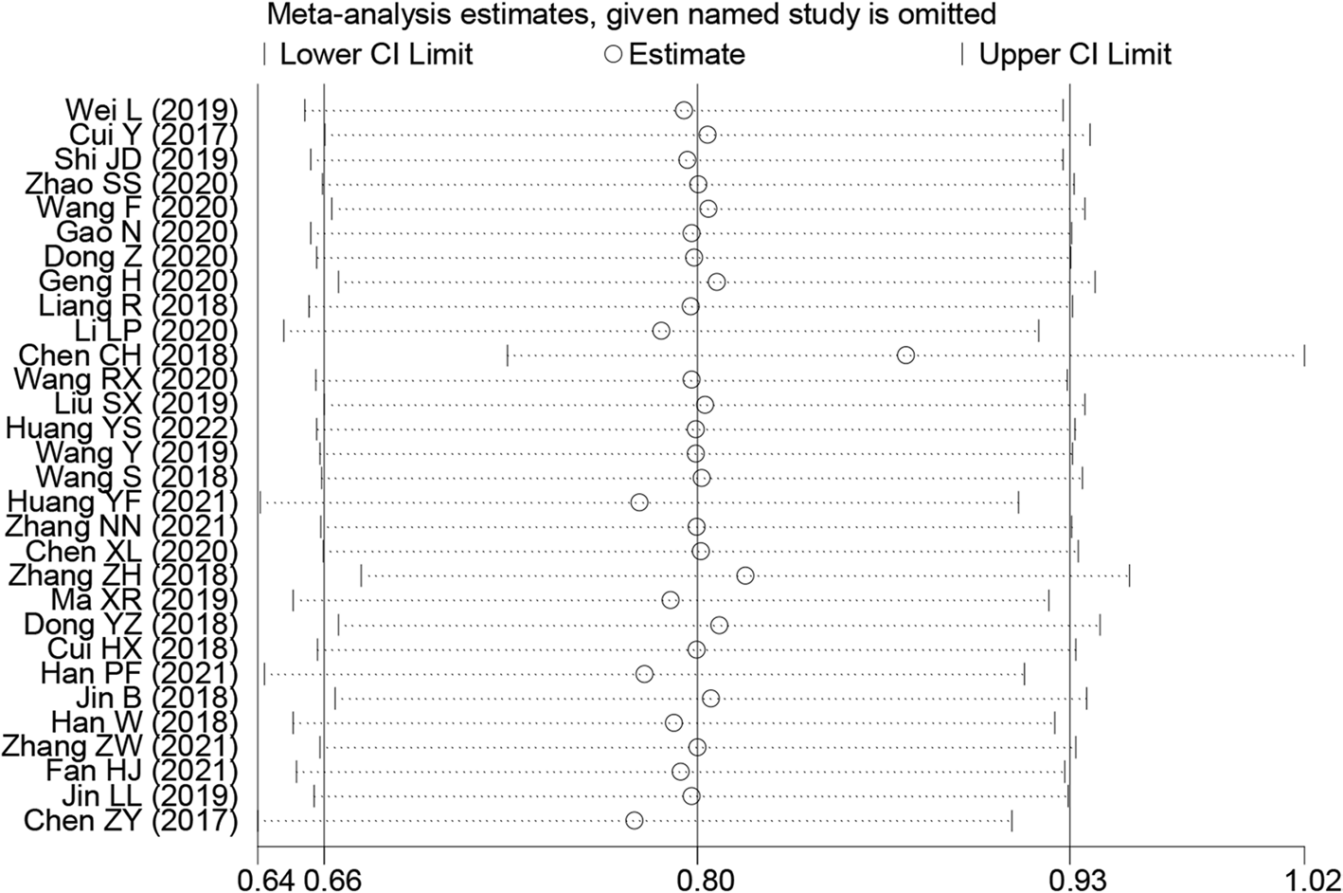
Sensitivity analysis for SNHG expression in detecting the overall survival (OS) of lung cancer patients. Note: HR: hazard ratio, CI: confidence interval

**Fig. 9:**
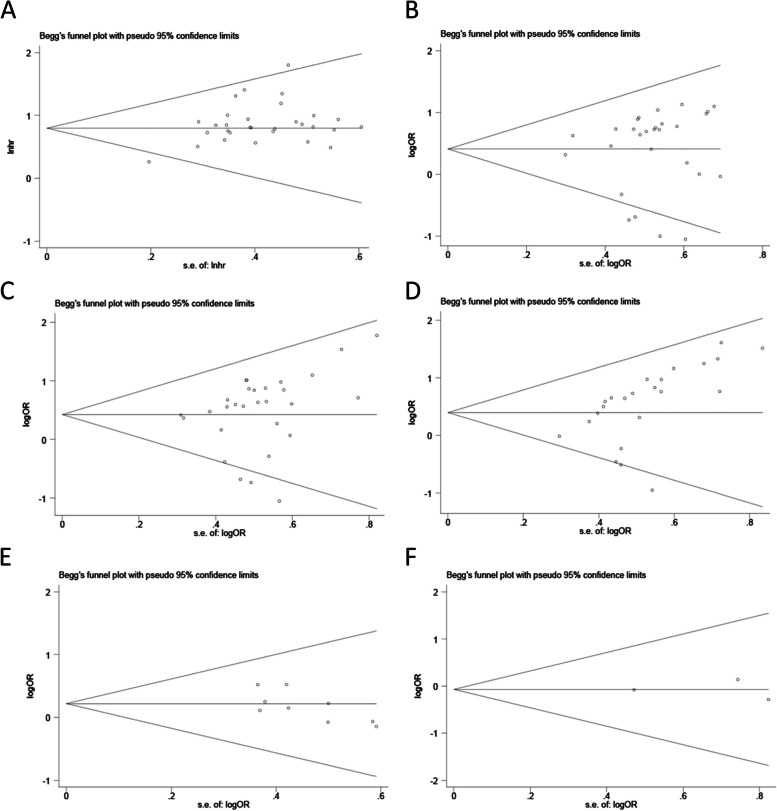
Publication bias regarding the relationship between SNHG expression and survival outcome in lung cancer. Note: **A** OS; **B** TNM stage; **C** LNM; **D** Tumor size; **E** Histological grade; **F** DM

The results of the sensitivity analysis showed that removing any one study did not significantly change the overall results, supporting the reliability and stability of this meta-analysis (Fig. 8). The results of publication bias analysis showed that there was no significant publication bias for each outcome, suggesting that none of the individual studies contribute significant statistical bias or other types of bias (Fig. 9).

## Discussion

An increasing number of long noncoding RNAs have been shown to be significantly involved in cancer progression and are clearly associated with cancer prognosis. Many noncoding RNAs have been reported to affect the occurrence and development of cancer by affecting cell biological behaviors such as tumor cell proliferation, migration, invasion, apoptosis, drug resistance or immune escape. In recent years, lung cancer-associated long noncoding RNAs have been gradually revealed. The biological behavior of lung cancer cells is significantly regulated by long noncoding RNAs. For example, Xu et al. reported that linc00473 may contribute to the proliferation, migration, invasion and inhibition of apoptosis of NSCLC cells by activating the ERK/p38 and MAPK signaling axes by sponging and downregulating miR-497-5p [17]. Zhong et al. revealed that lncRNA TTN-AS1 enhances the invasion and migration of NSCLC cells by increasing ZEB1 expression by suppressing miR-4677-3p [61]. Moreover, the prognosis of lung cancer patients has been reported to be significantly correlated with dysregulation of long noncoding RNAs [62].

The SNHG long noncoding RNA family, a class of small molecules without protein coding function, includes dozens of family members. An increasing number of studies have reported that the expression of SNHGs is dysregulated in lung tissue. Abnormally expressed SNHGs can affect the occurrence and development of lung cancer by affecting a series of biological behaviors of lung cancer cells, such as proliferation, migration, apoptosis, immune escape and drug resistance. Differential expression of SNHGs is significantly correlated with the prognosis of lung cancer. In this study, by pooling HR values from different studies, high expression of SNHGs was found to be positively correlated with a poor prognosis for lung cancer. SNHG2, SNHG3, and SNHG10 are expressed at low levels in lung cancer tissues, while other SNHGs are upregulated in lung cancer tissues. Considering these findings, we conducted a subgroup analysis based on the expression level of SNHGs in lung cancer, and the results showed that low expression of SNHG2 and SNHG3 predicted a poor prognosis for lung cancer, while high expression predicted a poor prognosis for lung cancer. Due to different cutoff values (mean and median), different analysis methods (univariate analysis and multivariate analysis), and different follow-up times and sample sizes among different studies, we performed subgroup analysis to compare the results of these types of studies. The results showed that among studies employing multivariate analysis, univariate analysis, the mean cutoff value and the median cutoff value, high expression of SNHG predicted poor prognosis of lung cancer. In addition, the combined HR value results showed that high expression of SNHG was significantly positively correlated with unsatisfactory progression-free survival and disease-free survival. The pooled OR value and 95% CI results showed that high expression of SNHG predicted advanced TNM stage, increased risk of lymph node metastasis and distant metastasis, larger tumor diameter, and worse histological grade. Considering the differences in research quality and inconsistent cutoff values among original studies, we also conducted subgroup analysis, and the results showed that high expression of SNHG still predicted poor survival outcomes among different subgroups.

An increasing number of studies have reported the molecular biological mechanism by which SNHG affects the progression of lung cancer (Table 5). First, SNHG can directly act on downstream genes or signaling pathways to affect a series of biological behaviors of lung cancer cells; for example, Zhang et al. reported that SNHG1 may contribute to the migration and invasion of NSCLC cells by upregulating zinc finger E-box-binding homeobox 1 (ZEB1) [56]. Shi et al. revealed that SNHG3 could drive the proliferation and migration of lung cancer cells by interacting with the IL-6/JAK2/STAT3 pathway [49]. Guo et al. discovered that DANCR (also named SNHG13) facilitates the proliferation, migration, invasion and EMT process of tumor cells through the upregulation of the p21 gene [34]. Second, SNHG could serve as a competing endogenous RNA and indirectly regulate downstream genes or signaling pathways by sponging microRNAs. For instance, Cui et al. suggested that SNHG1 may induce the proliferation and cell cycle and suppress the apoptosis of lung cancer cells by upregulating Wnt/β-catenin signaling by sponging and downregulating miR-101-3p [28]. Zhao et al. demonstrated that SNHG3 may promote the proliferation, migration, and invasion and inhibit the apoptosis of NSCLC cells through the upregulation of nuclear factor IX (NFIX) by sponging miR-1343-3p [21]. Wang et al. demonstrated that SNHG12 could facilitate the migration and EMT process of tumor cells by interacting with the Slug/ZEB2 signaling pathway by decreasing miR-218 [20]. In addition, some family members of SNHG can also regulate the drug resistance of NSCLC cells; for example, Wei et al. revealed that SNHG1 may reduce the cisplatin sensitivity of A549/DDP cells by increasing Rho-associated coiled-coil containing protein kinase 2 (ROCK2) expression by sponging and downregulating miR-101-3p [54]. Yang et al. reported that growth arrest specific 5 (GAS5, also named SNHG2) could reduce H1299/DDP cell migration, invasion and EMT and reduce cisplatin resistance through the upregulation of phospholysine phosphohistidine inorganic pyrophosphate phosphatase (LHPP) by sponging miR-217 [55]. Wang et al. discovered that SNHG5 was downregulated in NSCLC tissues, and high SNHG5 expression may enhance the sensitivity of A549 cells to gefitinib by interacting with the miR377/CASP1 axis [63]. In addition, SNHG may interfere with the immune escape of lung cancer cells; for instance, Huang et al. revealed that SNHG12 facilitates the immune escape of H1299 cells by interacting with the HuR/PD-L1/USP8 axis [37].

**Table 5:** Regulation mechanism of SNHG involved in lung cancers

Lnc RNA	Expression level	Role	micro-RNA	Downstream targets or pathways	Cell line	Function (high snhg expression)	Reference
SNHG1	upregulated	oncogene	miR-101-3p	ROCK2	A549, A549/DDP,NCI-H520/DDP	reduce cisplatin sensitivity	Wei L 2019 [54]
SNHG1	upregulated	oncogene	miR-101-3p	Wnt/β-catenin signaling	A549, SPC-A1, H23 and NCI-H520	promote proliferation and cell cycle, suppress apoptosis	Cui Y 2017 [28]
SNHG1	upregulated	oncogene	miR-361-3p	FRAT1	BEAS-2B, H23, H1299	induce cell proliferation, migration,invasion, inhibit apoptosis	Li XM 2020 [42]
SNHG1	upregulated	oncogene	-	ZEB1	H-266 and SK-MES-1	promotes cell metastasis and invasion	Zhang HY 2017 [56]
GAS5	downregulated	tumor suppressor gene	miR-217	LHPP	H1299, A549, A549/DDP and H1299/DDP	reduced NSCLC/DDP cell migration, invasion and EMT process, reduces cisplatin-resistance	Yang XH 2021 [55]
SNHG3	downregulated	tumor suppressor gene	miR-890	-	HBE, A549, H1299, H1975	Inhibits the Proliferation, Migration and Invasion, and Promotes the Apoptosis	Kang BJ 2021 [40]
SNHG3	upregulated	oncogene	miR-1343-3p	NFIX	BEAS-2B, H1299, H358, A549 and H1975	promotes proliferation, migration and invasion and inhibits apoptosis	Zhao LJ 2021 [60]
SNHG3	upregulated	oncogene	-	IL‐6/JAK2/STAT3 pathway	CMT‐167, LLC, CMT‐170, and CMT‐181	promotes proliferation and migration	Shi JD 2019 [49]
SNHG3	upregulated	oncogene	miR-216a	ZEB1	A549, H322, H1299, GLC-82, and SPC-A1	induce Proliferation, Migration and Invasion, and suppress Apoptosis	Zhao SS 2020 [21]
SNHG4	upregulated	oncogene	miR-let-7e	KDM3A/p21 pathway	H1299, H1650, H1975, and SPCA1	promotes proliferation, migration and invasion and inhibits apoptosis	Wang F 2020 [50]
SNHG5	downregulated	tumor suppressor gene	miR-377	miR377/CASP1 axis	PC9 and A549	enhances gefitinib sensitivity	Wang ZX 2018 [63]
SNHG6	upregulated	oncogene	miR-485-3p	VPS45	BEAS-2B, H520, H596, H1650, H1703	induce cell growth, migration and invasion	Gao N 2020 [32]
SNHG6	upregulated	oncogene	miR-490-3p	RSF1, Bcl-2, bax, caspase-3	A549, H460 and H1299	Promotes Proliferation and Inhibits Apoptosis	Dong Z 2020 [30]
SNHG6	upregulated	oncogene	-	ETS1, MMP2, MMP9	A549, H226, H292, ANP973 and H1299	promotes proliferation and migration	Geng H 2020 [33]
SNHG6	upregulated	oncogene	miR-26a-5p	E2F7	A549, H1299, H460,HCC827, NCl-H358 and NCl-H1650	promotes cell proliferation, migration, invasion, and EMT and induces cell cycle progression	Liang R 2018 [44]
SNHG7	upregulated	oncogene	miR-449a	miR-449a/TGIF2 axis	BEAS-2B, A549 and H1299	contributes to cell proliferation, migration, invasion and EMT process	Pang LL 2019 [48]
SNHG7	upregulated	oncogene	miR-181a-5p	AKT/mTOR Signaling Pathway	A549, NCI-H1299	Accelerates Proliferation, Migration and Invasion	Li LP 2020 [41]
SNHG8	upregulated	oncogene	miR-542-3p	CCND1/CDK6	(A549, H23, SPC-A1, and NCI-H292	contributes to cell proliferation	Chen CH 2018 [24]
SNHG9	upregulated	oncogene	-	CAPRIN1	BEAS-2B, SK-MES-1, H460, A549 and H1299	promoted DDP resistance	Wang RX 2020 [51]
SNHG10	downregulated	tumor suppressor gene	miR-21	-	KLN 205, HCC827	increasing SNHG10 expression suppress cell proliferation	Liang M 2020 [64]
SNHG10	downregulated	tumor suppressor gene	miR-543	SIRT1	H1581 and H1703	increasing SNHG10 expression suppress cell proliferation	Zhang Z 2020 [65]
SNHG11	upregulated	oncogene	miR‐4436a	Wnt/β‐catenin signaling pathway	A549, H1299, H460	facilitated lung cancer cell prol iferation, migration, invasion, and EMT process while suppressed cell apoptosis	Liu SX 2019 [46]
SNHG12	upregulated	oncogene	-	HuR/PD-L1/USP8 axis	A549, SW1573, H1975, H1299	induce immune escape	Huang YS 2022 [37]
SNHG12	upregulated	oncogene	miR-218	Slug/ZEB2 signaling pathway	A549, H1299	Accelerates Migration and EMT process	Wang Y 2019 [20]
SNHG13	upregulated	oncogene	miR-758-3p	-	SPC-A1 and NCI-H1299	promotes tumor NSCLC cell migration and invasion	Wang S 2018 [52]
SNHG13	upregulated	oncogene	-	p21	A549, H1299 and H358	contributes to cell proliferation, migration, invasion and EMT process	Guo LF 2019 [34]
SNHG13	upregulated	oncogene	miR-1225-3p	ErbB2	A549, SPCA1, H1299 and H1975	Enhanced Migration and Invasion	Huang YF 2021 [38]
SNHG13	upregulated	oncogene	-	HMGA2	SPCA1 and A549	promotes invasion	Zhang NN 2021 [57]
SNHG14	upregulated	oncogene	miR-382-5p	SPIN1	H1299, A549	induce Proliferation, Migration and Invasion, and suppress Apoptosis	Chen XL 2020 [25]
SNHG14	upregulated	oncogene	miR-340	-	16HBE, A549, NCI-H1975, NCI-H1299, SK-MES-1	induce Proliferation and suppress Apoptosis	Zhang ZH 2018 [58]
SNHG15	upregulated	oncogene	miR-211-3p	ZNF217	HBEC3, H358, H1299, H23 and A549	Promoted Proliferation and Migration	Ma XR 2019 [47]
SNHG15	upregulated	oncogene	miR-211-3p	-	H1799 and A549	promotes proliferation and migration	Cui HX 2018 [27]
SNHG15	upregulated	oncogene	miR-486	CDK14	A549, H460, SK-MES-1, and Calu-3	induce Proliferation, Migration and Invasion, and suppress Apoptosis	Jin B 2018 [39]
SNHG16	upregulated	oncogene	-	ALDH2, Bax, Bcl-2	A549 and SK-LU-1	cell proliferation	Li Y 2022 [43]
SNHG16	upregulated	oncogene	miR-146a	MUC5AC	A549, NCI-H292, NCI-H460, and NCI-H1703	promotes NSCLC cell proliferation, migration and invasion	Han W 2018 [36]
SNHG17	upregulated	oncogene	miR-193a-5p	NETO2	BEAS-2B, A549, H1299, H1650, H1975 and CALU-3	facilitate migration, invasion, proliferation and EMT	Zhang ZW 2021 [59]
SNHG18	upregulated	oncogene	miR-211-5p	miR-211-5p/BRD4 axis	A549, H1299, H23, H460, and H1792	promotes NSCLC cell proliferation, migration and invasion	Fan HJ 2021 [31]
SNHG20	upregulated	oncogene	miR-154	ZEB2 and RUNX2	A549, H322, H1299, GLC-82, and SPC-A1	promotes proliferation, migration and invasion, and suppresses apoptosis	Jin LL 2019 [45]
SNHG20	upregulated	oncogene	-	P21	PC9, SPC-A1, NCIH1975, H1299 and A549	promotes cell proliferation and migration	Chen ZY 2017 [26]
SNHG20	upregulated	oncogene	miR-342	DDX49	BEAS-2B, A549 and H1299	promoted proliferation, invasion and inhibited cell apoptosis	Wang XL 2020 [53]

*ROCK2* Rho-associated coiled-coil containing protein kinase 2, *FRAT1* FRAT Regulator Of WNT Signaling Pathway 1, *ZEB1* Zinc finger E-box-binding homeobox protein 1, *LHPP* Phospholysine Phosphohistidine Inorganic Pyrophosphate Phosphatase, *NFIX* Nuclear Factor I X, *JAK2* Janus kinase 2, *KDM3A* Lysine Demethylase 3A, *CASP1* Caspase 1, *VPS45* Vacuolar Protein Sorting 45 Homolog, *RSF1* Remodeling And Spacing Factor 1, *MMP2* Matrix metalloproteinase 2, *MMP9* Matrix metalloproteinase 9, *E2F7* E2F Transcription Factor 7, *TGIF2* TGFB Induced Factor Homeobox 2, *CCND1* Cyclin D1, *CDK6* Cyclin-dependent kinase 6, *SIRT1* Sirtuin 1, *ErbB2* Human epidermal growth factor receptor 2, *HMGA2* High Mobility Group AT-Hook 2, *SPIN1* Spindlin 1, *ZNF217* Zinc Finger Protein 217, *CDK14* Cyclin-dependent kinase 14, *ALDH2* Acetaldehyde dehydrogenase 2, *MUC5AC* Mucin 5AC, *NETO2* Neuropilin And Tolloid Like 2, *ZEB2* Zinc finger E-box binding homeobox (Zeb) 2, *RUNX2* Runt-related transcription factor 2, *DDX49* DEAD-Box Helicase 49

There are inevitably some limitations to this study. First, all the included studies were from China, so the conclusions of this study may only be applicable to patients in China or East Asia. Second, among all the included original studies, some studies directly provided HR values, while others only provided survival curves. We could only obtain HR values indirectly through Enguage software, which makes the combined OS value of this study somewhat inaccurate. statistical bias. However, this study is the first meta-analysis to explore the correlation between the expression level of the SNHG family and the prognosis of lung cancer. In addition, this study comprehensively summarizes the molecular biological mechanism of each member of the SNHG family affecting the occurrence and development of lung cancer.

## Conclusion

Most SNHGs are upregulated in lung cancer, and only some SNHGs are downregulated in lung cancer. High SNHG expression predicts poor overall survival and disease-free survival in lung cancer. SNHG may be a potential prognostic marker and a promising therapeutic target.

### Supplementary Information

**Additional file 1. **

## Data Availability

All data generated or analyzed during this study are included in this published article or are available from the corresponding author upon reasonable request.
