# Supplementary material for: High SNHG expression may predict a poor lung cancer prognosis based on a meta-analysis
Source: BMC Cancer. 2023 Dec 16;23:1243. doi: 10.1186/s12885-023-11706-4 (PMC10725607; doi:10.1186/s12885-023-11706-4)
Supplement: Supplementary file 1 — Additional file 1. [file 12885_2023_11706_MOESM1_ESM.docx]

| **Data Sharing Statement** | | |
| --- | --- | --- |
| **Article Info** | n/a (editor will fill in this) | |
| **Item** | **Question** | **Authors’ Response （place “-” if not applicable）** |
| 1 | Would you like to share data collected for your study to others? | Yes |
| 2 | If not, would you like to share the reason for your decision? | **-** |
| 3 | What data in particular will be shared? | All raw data |
| 4 | Any other documents will be share? Such as study protocol, statistical analysis plan, informed consent form, clinical study report, analytic code. | Yes |
| 5 | When will data availability begin? | After accepted and published |
| 6 | When will data availability end? | The data will always be open without deadline |
| 7 | To whom will you share the data? | For non-commercial use, we will give the original data |
| 8 | For what type of analysis or purpose? | scientific research |
| 9 | How or where can the data/documents be obtained? | All data generated or analyzed during this study are included in this published article or are available from the corresponding author on reasonable request. |
| 10 | Any other restrictions? | no |
